# Epigenetic associations of *GPNMB* rs199347 variant with alcohol consumption in Parkinson’s disease

**DOI:** 10.3389/fpsyt.2024.1377403

**Published:** 2024-07-18

**Authors:** Yen-Chung Chen, Yi-Chia Liaw, Oswald Ndi Nfor, Chih-Hsuan Hsiao, Ji-Han Zhong, Shey-Lin Wu, Yung-Po Liaw

**Affiliations:** ^1^ Department of Public Health and Institute of Public Health, Chung Shan Medical University, Taichung, Taiwan; ^2^ Department of Neurology, Changhua Christian Hospital, Changhua, Taiwan; ^3^ Neurological Institute, Taipei Veterans General Hospital, Taipei, Taiwan; ^4^ Department of Electrical Engineering, National Changhua University of Education, Changhua, Taiwan; ^5^ Department of Medical Imaging, Chung Shan Medical University Hospital, Taichung, Taiwan; ^6^ Institute of Medicine, Chung Shan Medical University, Taichung, Taiwan

**Keywords:** Parkinson disease, DNA methylation, epigenesis, genetic, alcohol-related disorders, GPNMB

## Abstract

**Introduction:**

Alcohol consumption can induce a neuroinflammatory response and contribute to the progression of neurodegeneration. However, its association with Parkinson’s disease (PD), the second most common neurodegenerative disorder, remains undetermined. Recent studies suggest that the glycoprotein non-metastatic melanoma protein B (GPNMB) is a potential biomarker for PD. We evaluated the association of rs199347, a variant of the *GPNMB* gene, with alcohol consumption and methylation upstream of *GPNMB*.

**Methods:**

We retrieved genetic and DNA methylation data obtained from participants enrolled in the Taiwan Biobank (TWB) between 2008 and 2016. After excluding individuals with incomplete or missing information about potential PD risk factors, we included 1,357 participants in our final analyses. We used multiple linear regression to assess the association of *GPNMB* rs199347 and chronic alcohol consumption (and other potential risk factors) with *GPNMB* cg17274742 methylation.

**Results:**

There was no difference between the distribution of *GPNMB* rs199347 genotypes between chronic alcohol consumers and the other study participants. A significant interaction was observed between the GPNMB *rs199347* variant and alcohol consumption (p = 0.0102) concerning cg17274742 methylation. Compared to non-chronic alcohol consumers with the AA genotype, alcohol drinkers with the rs199347 GG genotype had significantly lower levels (hypomethylation) of cg17274742 (p = 0.0187).

**Conclusion:**

Alcohol consumption among individuals with the rs199347 GG genotype was associated with lower levels of cg17274742 methylation, which could increase expression of the *GPNMB* gene, an important neuroinflammatory-related risk gene for PD.

## Introduction

Parkinson’s disease (PD) is the second most common neurodegenerative disorder, affecting >1% of the worldwide population ≥65 years of age, and is characterized primarily by slow movement, tremors, and rigidity (1, 2). The etiology of PD remains unknown, although it is believed to result from a combination of genetic, environmental, and behavioral factors by progressive loss of dopaminergic neurons in the substantia nigra and accumulation of α-synuclein within Lewy bodies (3–5). Pathophysiologically, PD has been associated with α-synuclein misfolding and aggregation, mitochondrial dysfunction, impairment of protein clearance, neuroinflammation, and oxidative stress (6–8). Epigenetic modifications, which can connect environment or behavior factors with the genetic changes underlying disease (9, 10), have been shown to modulate the immune systems of patients with PD (11–22).

Glycoprotein non-metastatic melanoma protein B (GPNMB), a type I transmembrane protein involved in immune cell maturation and activation (23), has been shown to be a promising biomarker of PD risk (24–27). *GPNMB* is highly expressed in macrophages and microglia, which play a significant role in neuroinflammation, and was shown to be upregulated in disease-associated microglia in neurodegenerative animal models (28, 29). A prominent single-nucleotide polymorphism (SNP) of *GPNMB*, rs199347, plays a significant role in modulating systemic immune response, especially neuroinflammation (21–26, 30). The rs199347 variant genotype and minor allele involves a guanine instead of the reference alanine (31) and has been identified as highly associated with PD risk (24). Recently, GPNMB was shown to promote the toxic aggregation of the alpha-synuclein protein in substantia nigra, which is believed to contribute to neurodegeneration, and could be a potential target for PD treatment (24, 32). Furthermore, recent proteomic analyses of cerebrospinal fluid have identified GPNMB as a primary causal protein in PD emphasizing its role in the disease’s heterogeneity and causality (33). Notably, studies involving animal models have demonstrated that overexpression of GPNMB can mitigate degeneration of dopaminergic neurons and provide anti-neuroinflammatory benefits (34, 35).The differences among the rs199347 genotypes (AA, AG, and GG) are significantly associated with *GPNMB* expression in the brain as well as whole blood (24, 25, 27, 36–39). Moreover, large, integrated GWAS on methylation data from brain samples of patients with PD found that the association between *GPNMB* and PD could be regulated by DNA methylation (25, 27, 39). Indeed, hypomethylation at cg17274742, which is proximal to the 7p15 chromosomal region in which *GPNMB* is located, is associated with increased *GPNMB* expression in PD patients (25, 27).

Alcohol consumption can affect epigenetic modification and gene expression (40–42), and associated DNA methylation changes have been observed in both the peripheral and central nervous systems (43, 44). Chronic alcohol consumption can trigger neuroinflammation resulting in central nervous system injury and possibly neurodegeneration (45–48). High alcohol consumption has been shown to increase *GPNMB* levels (49, 50) suggesting an involvement with PD. However, the connection between alcohol drinking and PD remains poorly understood, and epidemiological studies show contradictory data (51–55). Whether PD is caused by chronic alcohol consumption remains unclear (53–56), although chronic alcohol consumption has been found to drive biological mechanisms with significant changes to DNA methylation in serum and brain tissues (40, 41, 43, 44).

To date, no study has evaluated the role of alcohol intake on the expression of *GPNMB*, and specifically the PD-associated *GPNMB* SNP rs199347, and DNA methylation. Identifying methylation patterns associated with alcohol consumption and *GPNMB* expression could help elucidate the influence of alcohol consumption on the risk of PD.

## Methods

### Participants and data source

Data were obtained from the Taiwan Biobank (TWB), an ongoing prospective cohort study of more than 150,000 participants. The TWB contains demographic and whole-genome sequencing data of Taiwanese (99% Han Chinese) without cancer aged 20 to 70 years, with lifestyle information captured through individual interviews (57, 58).

We enrolled all 2,352 individuals aged 20–70 years in the TWB with DNA methylation data. Using previously published epidemiology studies (59–63), we developed a list of potential risk factors for PD as variables for consideration: sex, age, body mass index, cigarette smoking, alcohol drinking, exercise, coffee drinking, uric acid levels, and hypertension. We excluded 995 individuals with incomplete or missing information, and the remaining 1,357 participants with complete information were included in our final analysis.

This study was approved by the Institutional Review Board of Chung Shan Medical University Hospital (CS1–20009).

### DNA methylation assessment

The TWB assessed DNA methylation from whole blood using the Infinium MethylationEPIC BeadChip Kit (Illumina Inc, San Diego, CA, USA) (64–66). Cell-type heterogeneity was adjusted using the Reference-Free Adjustment for Cell-Type composition (ReFACTor) method (67). Methylation levels were quantified using beta values (0–1), which roughly correspond to the percent methylation at a particular site.

### Genetic variant assessment

Genotyping was performed at Academia Sinica in Taiwan using a customized Axiom Genome-Wide Array Plate (Affymetrix, Santa Clara, CA, USA), and SNP information was obtained after imputation methods (68). All SNPs with minor allele frequency (MAF) <1% were excluded.

### Covariate analysis

Chronic alcohol consumption was defined as drinking more than 150 ml of alcohol-containing beverage(s) per week for ≥6 months prior to enrollment in the TWB. Body mass index (BMI) was calculated (kg/m^2^) and categorized as underweight (BMI < 18.5), normal weight (18.5 ≤ BMI < 24), overweight (24 ≤ BMI < 27), and obese (BMI ≥ 27). Current cigarette smokers were those who smoked continuously for ≥6 months, and former smokers were those who previously smoked but quit for ≥6 months. Participants who exercised were those reporting a regular habit of exercising ≥30 min each three times per week. Hypertension was classified by patients self-reporting a diagnosis to a physician.

### Statistical analysis

We used multiple linear regression to evaluate the association of *GPNMB* rs199347 and alcohol drinking with *GPNMB* cg17274742 methylation, as well as the interaction between *GPNMB* rs199347 and alcohol drinking. The differences between variables were calculated using a t-test for continuous variables and a chi-square test for categorical variables. Statistical significance was defined as α < 0.05. All analyses were conducted using PLINK version 1.9 beta (69) and SAS version 9.4 (SAS Institute Inc, Cary, NC, USA).

## Results

Overall, 1,357 participants comprising 648 men and 709 women were included in the study (**Table 1**). The mean *GPNMB* cg17274742 methylation levels (beta values) for individuals with chronic alcohol consumption was 0.9484 ± 0.0009 (mean ± standard error), compared to 0.9484 ± 0.0003 for those who did not chronically consume alcohol. For the non-chronic group, the number (percentage) of participants with the *GPNMB* rs199347 AA, AG, and GG genotypes was 633 (51.72%), 511 (41.75%), 80 (6.54%), respectively. For the chronic alcohol-drinking group, the number (percentage) with the GPNMB rs199347 AA, AG, and GG genotypes were 66 (49.62%), 53 (39.85%), and 14 (10.53%), respectively. The methylation levels and genotype distribution did not differ significantly between the chronic alcohol consumers and the other study participants.

**Table 1:** Demographic characteristics of the study participants.

	Control group (n = 1,224)	Chronic alcohol consumers (n = 133)	p-Value
**Beta value of cg17274742 methylation** (mean ± SE)	0.9484 ± 0.000319	0.9484 ± 0.000935	0.9739
**GPNMB rs199347** (n, %)			0.2272
AA	633 (51.72)	66 (49.62)	
AG	511 (41.75)	53 (39.85)	
GG	80 (6.54)	14 (10.53)	
**Sex** (n, %)			**<0.0001**
Female	693 (56.62)	16 (12.03)	
Male	531 (43.38)	117 (87.97)	
**Age** (years)	49.1 ± 0.3	51.6 ± 0.9	0.0127
**Body mass index** (n, %)			**<0.0001**
Normal weight	611 (49.92)	41 (30.83)	
Underweight	37 (3.02)	1 (0.75)	
Overweight	339 (27.70)	57 (42.86)	
Obesity	237 (19.36)	34 (25.56)	
**Cigarette smoking** (n, %)			**<0.0001**
Never	978 (79.90)	50 (37.59)	
Former	147 (12.01)	43 (32.33)	
Current	99 (8.09)	40 (30.08)	
**Exercise** (n, %)			0.9680
No	688 (56.21)	75 (56.39)	
Yes	536 (43.79)	58 (43.61)	
**Coffee intake** (n, %)			**0.0152**
No	793 (64.79)	72 (54.14)	
Yes	431 (35.21)	61 (45.86)	
**Uric acid level** (mg/dl)	5.4238 ± 0.0396	6.4609 ± 0.1343	**<0.0001**
**Hypertension** (n, %)			**0.0060**
No	1078 (88.07)	106 (79.70)	
Yes	146 (11.93)	27 (20.30)	

Continuous data are displayed as mean ± standard error (SE) and categorical data as numbers (percentages). Bold values means p value <0.05.

The *GPNMB* rs199347 variant and alcohol consumption was not significantly associated with the methylation of cg17274742 (**Table 2**). However, men had significantly lower levels of cg17274742 methylation compared to women (β = −0.00302, p = 0.0169), while hypertensive patients had significantly higher levels of cg17274742 methylation compared to non-hypertensive patients (β = 0.00208, p = 0.0171).

**Table 2:** Association of rs199347 and alcohol consumption with cg17274742 methylation.

	β	p-Value
GPNMB rs199347 (ref: AA)		
AG	0.00067276	0.2425
GG	0.00047793	0.6683
Chronic alcohol consumption (ref: no)		
Yes	−0.00042063	0.6713
Sex (ref: female)		
Male	−0.00302	**0.0169**
**Age** (years)	0.00005373	0.1891
Body mass index (ref: normal weight)		
Underweight	−0.00305	0.0742
Overweight	0.00046711	0.4880
Obesity	−0.00052500	0.5004
Cigarette smoking (ref: never)		
Former	0.00002321	0.9788
Current	−0.00005172	0.9594
Exercise (ref: no)		
Yes	−0.00105	0.0839
Coffee intake (ref: no)		
Yes	0.00044856	0.4366
**Uric acid level** (mg/dl)	0.00011273	0.6367
Hypertension (ref: no)		
Yes	0.00208	**0.0171**

β, beta coefficient. Bold values means p value <0.05.

*GPNMB* rs199347 variant and alcohol drinking had a significant interaction (p = 0.0102) with the methylation of cg17274742 (**Table 3**). After stratifying by alcohol consumption, rs199347 was not significantly associated with the methylation of cg17274742 in the control group. However, compared to the rs199347 AA genotype, the GG genotype was significantly associated with lower levels of methylation at cg17274742 (β = −0.00635, p *=* 0.0366).

**Table 3:** Association of rs199347 with methylation of cg17274742 stratified by alcohol consumption.

	Control group (n = 1,224)		Chronic alcohol consumers (n = 133)	
	β	p-Value	β	p-Value
GPNMB rs199347 (ref: AA)				
AG	0.00081125	0.1804	-0.00152	0.4276
GG	0.00186	0.1236	-0.00635	**0.0366**
Sex (ref: female)				
Male	−0.00258	**0.0497**	−0.01076	**0.0488**
**Age** (years)	0.00006865	0.1141	−0.00007678	0.5481
Body mass index (ref: normal weight)				
Underweight	−0.00338	0.0513	0.01736	0.1111
Overweight	0.00031558	0.6586	0.00313	0.1606
Obesity	−0.00065127	0.4299	0.00065871	0.7934
Cigarette smoking (ref: never)				
Former	−0.00025122	0.7933	0.00356	0.1233
Current	−0.00008491	0.9409	0.00169	0.4856
Exercise (ref: no)				
Yes	−0.00107	0.0967	−0.00296	0.1258
Coffee intake (ref: no)				
Yes	0.00034271	0.5748	0.00266	0.1489
**Uric acid level** (mg/dl)	0.00017402	0.4992	0.0000587	0.9315
Hypertension (ref: no)				
Yes	0.00258	0.0061	−0.00044093	0.8616

Interaction (rs199347 * Alcohol consumption) p-value = 0.0102.

β, beta coefficient. Bold values means p value <0.05.

After combining the rs199347 genotypes and alcohol consumption (**Table 4**), cg17274742 was significantly hypomethylated among chronic alcohol drinkers with the rs199347 GG genotype compared to the control group with the AA genotype (β= −0.00654, p = 0.0187).

**Table 4:** Cg17274742 methylation based on rs199347 and alcohol consumption.

	β	*p*-Value
GPNMB rs199347 and alcohol consumption (ref: AA and non-chronic alcohol consumers/control)		
AG and control	0.00080588	0.1813
GG and control	0.0018	0.1343
AA and chronic alcohol consumer	0.00108	0.4274
AG and chronic alcohol consumer	0.00039023	0.7921
GG and chronic alcohol consumer	−0.00654	**0.0187**
Sex (ref: female)		
Male	−0.00299	**0.0178**
**Age** (years)	0.00005587	0.1711
Body mass index (ref: normal weight)		
Underweight	−0.003	0.0784
Overweight	0.00048126	0.4739
Obesity	−0.00061427	0.4298
Cigarette Smoking (ref: never)		
Former	0.00003035	0.9722
Current	−0.0001492	0.8831
Exercise (ref: no)		
Yes	−0.0011	0.0698
Coffee Intake (ref: no)		
Yes	0.00046963	0.4143
**Uric acid level** (mg/dl)	0.00016297	0.4950
Hypertension (ref: no)		
Yes	0.00219	**0.0119**

β, beta coefficient. Bold values means p value <0.05.

## Discussion

Using data from a large, national prospective cohort study, we identified an association between cg17274742 methylation status, the *GPNMB* rs199347 polymorphism, and alcohol consumption. Our findings suggest that carriers of the rs199347 GG genotype and those who chronically consume alcohol have significantly more cg17274742 hypomethylation, which may result in increased *GPNMB* expression. This is the first study to find a relationship between a specific *GPNMB* polymorphism and alcohol-associated methylation changes suggesting that a lifestyle change, particularly a reduction in alcohol consumption for carriers of the rs199347 GG genotype, could reduce *GPNMB* expression, which is highly correlated with the risk of PD.

Recent studies highlight the importance and benefits of involving diverse and multiethnic populations in genetic studies (70, 71), including more accurately representing the risks of genetically associated diseases in different populations (72). Most published GWAS and methylomic studies involving the *GPNMB* rs199347 variant were conducted in majority-white study populations (36–39, 73, 74). Our study uses information obtained from the Taiwan Biobank (TWB), the largest biobank in East Asia with dense SNP array data, and leverages its high-coverage whole-genome sequencing and DNA methylation data in a population of Han Chinese (58). Notably, the rs199347 genotype frequencies observed in our study (A = 0.72, G = 0.28) are roughly equivalent to those reported by the Allele Frequency Aggregator Project in East Asia (A = 0.716, G = 0.283), which is different from the frequencies reported in white individuals (A = 0.59, G = 0.41) (31). While the TWB provides a robust dataset for understanding genetic associations in the Han Chinese population, it is crucial to discuss the generalizability of our findings beyond this group. The genetic and lifestyle diversity across different populations may impact the observed associations. Therefore, further studies are needed to explore how genetic differences between populations might influence the relationship between the GPNMB rs199347 variant, alcohol consumption, and Parkinson’s disease risk. Addressing these potential impacts would enhance the broader applicability of our findings and contribute to a more comprehensive understanding of genetic risk factors across diverse populations.

A novel integrative approach has been developed to align expression quantitative trait loci (eQTL) with genome-wide association study (GWAS) signals in Parkinson’s disease (PD), utilizing an updated PD GWAS dataset. This strategy highlighted the methylation site cg17274742 within the GPNMB gene, a site of interest for Coloc analysis, which investigates common causal variants shared between eQTL and GWAS data. Methylation at this specific locus not only affects gene expression but also influences splicing activities at the GPNMB/NUPL2 locus facilitated by robust protein–protein interactions. These interactions may connect to genes associated with either Mendelian or sporadic forms of PD (25). The presence of PD-associated variants at this methylation site reveals critical molecular pathways influencing the pathogenesis of PD, thereby emphasizing the significant role of both genetic and epigenetic factors in its development. In our study, we found that males had more cg17274742 hypomethylation, which may increase *GPNMB* expression. After correcting for age, the prevalence of PD in men is approximately 1.4 times higher than that in women (75), although a 2014 meta-analysis suggests that this difference is evident only in the 50- to 59-year age group (59). Additionally, we found that hypertension was associated with cg17274742 hypermethylation, which could decrease *GPNMB* expression and potentially reduce PD risk (24, 36). Published evidence connecting hypertension and PD diagnosis is contradictory (76–81), and different effects of hypertension have been observed in white versus Asian populations (76–79). Moreover, some antihypertensive medications may contribute to PD risk; inhibitors of the renin–angiotensin–aldosterone system may delay proinflammatory effects, and alpha-1-adrenergic receptor antagonists can enhance glycolysis and could reduce PD risk (82–87).

Chronic alcohol consumption can damage the central nervous system (45), and a recent translational study suggests a strong correlation between alcohol use disorder and the inflammatory response in the brain (45). Chronic alcohol consumption can trigger pro-inflammatory cytokines by activating peripheral macrophages and microglia in the central nervous system, which may alter the neuroinflammatory state in the brain (46, 47). Previous studies have noted the importance of different biological processes and functions of GPNMB, including cell differentiation and development, inflammation and immune response, progression, and neurodegeneration deterioration (23). Additionally, the Genotype-Tissue Expression (GTEx) project has identified that the GPNMB rs199347 variant significantly influences gene expression in various brain tissues, including the basal ganglia, cerebellum, and frontal cortex (88, 89). These regions are closely linked to neural mechanisms, particularly the dopaminergic pathways, which are crucial in the pathophysiology of PD (3, 6). The impact of this variant on these brain regions underscores its potential role in the clinical manifestations of PD. Our finding suggests that lifestyle changes, particularly a reduction in alcohol consumption for carriers of the rs199347 GG genotype, could modulate GPNMB expression, thereby identifying a potentially modifiable risk factor in Parkinson’s disease.

Our study was limited by the uncertainty around the amount of alcohol consumption. The TWB survey quantifies alcohol consumption in milliliters (ml), not milligrams (mg), and is therefore difficult to standardize across the study population. Additionally, data on alcohol consumption were self-reported and are subject to recall bias. Another limitation of our investigation concerns our inability to comprehensively account for all potential confounding variables, including dietary habits, lifestyle choices, and environmental exposures. Nevertheless, we mitigated this by controlling for a range of well-documented risk and protective factors, such as age, body mass index (BMI), smoking status, alcohol consumption, coffee intake, serum uric acid levels, and hypertension, all of which have been extensively studied in PD epidemiological research.

Ultimately, our results provide insight into the genetic and lifestyle factors associated with *GPNMB* expression, which is a potential biomarker and therapeutic target of PD. Future experiments using animal models or human cell lines to examine the underlying mechanisms behind this potential neuroinflammatory association in the central nervous system are warranted.

## Conclusion

In summary, we found that having the GG genotype of GPNMB rs199347 with drinking habits decreases GPNMB methylation among Taiwan Biobank participants. These results provide information on the genetic and lifestyle factors that contribute to the expression, which is a PD potential biomarker and therapeutic target of PD, and could be used as a reference for experimental, longitudinal, or intervention studies evaluating the disease and its associated variables and mechanisms.

## Data availability statement

The access to and use of the Taiwan Biobank data in the present work was approved by the Ethics and Governance Council (EGC) of Taiwan Biobank (approval number: TWBR10907-05) and the Institutional Review Board (IRB) of National Health Research Institutes, Taiwan (approval number: EC1090402-E). The data collection of Taiwan Biobank was approved by the Ethics and Governance Council (EGC) of Taiwan Biobank and the Department of Health and Welfare, Taiwan (Wei-Shu-I-Tzu NO.1010267471). The data utilized in this study are sourced from the Taiwan Biobank, accessible at (https://www.twbiobank.org.tw/). Due to restrictions, the data are not publicly available and were used under license specifically for this study. However, these data can be obtained from the corresponding author, Yung-Po Liaw, upon reasonable request and with permission from the Taiwan Biobank.

## Ethics statement

Our study involves patients enrolled in the Taiwan Biobank (TWB). TWB was approved by the Institutional Review Board on Biomedical Science Research/IRB-BM, Academia Sinica and by the Ethics and Governance Council of Taiwan Biobank, Taiwan. Written informed consent was obtained before data collection from each participant in accordance with institutional requirements and the principles of the Declaration of Helsinki. This study was approved by the Institutional Review Board of the Chung Shan Medical University Hospital (CS1–20009). Participants in the Taiwan Biobank provided their written informed consent during enrollment. All methods were performed according to the relevant guidelines and regulations.

## Author contributions

Y-CC: Conceptualization, Investigation, Validation, Visualization, Writing – original draft. Y-CL: Methodology, Validation, Writing – review & editing. ON: Investigation, Methodology, Supervision, Validation, Writing – review & editing. C-HH: Data curation, Formal analysis, Methodology, Writing – review & editing. J-HZ: Data curation, Formal analysis, Methodology, Writing – review & editing. S-LW: Conceptualization, Investigation, Validation, Writing – review & editing. Y-PL: Conceptualization, Funding acquisition, Investigation, Resources, Validation, Writing – review & editing.

## References

[1] Bergman H Deuschl G. Pathophysiology of Parkinson's disease: from clinical neurology to basic neuroscience and back. Mov Disord. (2002) 17 Suppl 3:S28–40. doi: 10.1002/(ISSN)1531-8257 11948753

[2] Postuma RB Berg D Stern M Poewe W Olanow CW Oertel W. MDS clinical diagnostic criteria for Parkinson's disease. Mov Disord. (2015) 30:1591–601. doi: 10.1002/mds.26424 26474316

[3] Balestrino R Schapira AHV. Parkinson disease. Eur J Neurol. (2020) 27:27–42. doi: 10.1111/ene.14108 31631455

[4] Alam M Schmidt WJ. Rotenone destroys dopaminergic neurons and induces parkinsonian symptoms in rats. Behav Brain Res. (2002) 136:317–24. doi: 10.1016/S0166-4328(02)00180-8 12385818

[5] Battaglia S Nazzi C Thayer JF. Genetic differences associated with dopamine and serotonin release mediate fear-induced bradycardia in the human brain. Trans Psychiatry. (2024) 14:24. doi: 10.1038/s41398-024-02737-x PMC10789820 38225222

[6] Jankovic J Tan EK. Parkinson's disease: etiopathogenesis and treatment. J Neurol Neurosurg Psychiatry. (2020) 91:795–808. doi: 10.1136/jnnp-2019-322338 32576618

[7] Surguchov A Surguchev A. Synucleins: new data on misfolding, aggregation and role in diseases. Biomedicines. (2022) 10:3241. doi: 10.3390/biomedicines10123241 36551997 PMC9775291

[8] Tanaka M Szabó Á Vécsei L Giménez-Llort L. Emerging translational research in neurological and psychiatric diseases: from *in vitro* to *in vivo* models. Int J Mol Sci. (2023) 24:15739. doi: 10.3390/ijms242115739 37958722 PMC10649796

[9] Jirtle RL Skinner MK. Environmental epigenomics and disease susceptibility. Nat Rev Genet. (2007) 8:253–62. doi: 10.1038/nrg2045 PMC5940010 17363974

[10] Tanaka M Szabó Á Vécsei L. Preclinical modeling in depression and anxiety: Current challenges and future research directions. Adv Clin Exp Med. (2023) 32:505–9. doi: 10.17219/acem/165944 37212773

[11] Scheiblich H Bousset L Schwartz S Griep A Latz E Melki R. Microglial NLRP3 inflammasome activation upon TLR2 and TLR5 ligation by distinct α-synuclein assemblies. J Immunol. (2021) 207:2143–54. doi: 10.4049/jimmunol.2100035 PMC8490941 34507948

[12] Tremblay M-E Cookson MR Civiero L. Glial phagocytic clearance in Parkinson’s disease. Mol Neurodegen. (2019) 14:16. doi: 10.1186/s13024-019-0314-8 PMC6451240 30953527

[13] Grozdanov V Bousset L Hoffmeister M Bliederhaeuser C Meier C Madiona K. Increased immune activation by pathologic α-synuclein in Parkinson's disease. Ann Neurol. (2019) 86:593–606. doi: 10.1002/ana.25557 31343083

[14] Tronel C Largeau B Santiago Ribeiro MJ Guilloteau D Dupont AC Arlicot N. Molecular targets for PET imaging of activated microglia: the current situation and future expectations. Int J Mol Sci. (2017) 18. doi: 10.3390/ijms18040802 PMC5412386 28398245

[15] Joers V Tansey MG Mulas G Carta AR. Microglial phenotypes in Parkinson's disease and animal models of the disease. Prog Neurobiol. (2017) 155:57–75. doi: 10.1016/j.pneurobio.2016.04.006 27107797 PMC5073045

[16] Grozdanov V Bliederhaeuser C Ruf WP Roth V Fundel-Clemens K Zondler L. Inflammatory dysregulation of blood monocytes in Parkinson's disease patients. Acta Neuropathol. (2014) 128:651–63. doi: 10.1007/s00401-014-1345-4 PMC4201759 25284487

[17] Sanchez-Guajardo V Barnum CJ Tansey MG Romero-Ramos M. Neuroimmunological processes in Parkinson's disease and their relation to α-synuclein: microglia as the referee between neuronal processes and peripheral immunity. ASN Neuro. (2013) 5:113–39. doi: 10.1042/AN20120066 PMC3639751 23506036

[18] Brochard V Combadière B Prigent A Laouar Y Perrin A Beray-Berthat V. Infiltration of CD4+ lymphocytes into the brain contributes to neurodegeneration in a mouse model of Parkinson disease. J Clin Invest. (2009) 119:182–92. doi: 10.1172/JCI36470 PMC2613467 19104149

[19] Gerhard A Pavese N Hotton G Turkheimer F Es M Hammers A. *In vivo* imaging of microglial activation with [11C](R)-PK11195 PET in idiopathic Parkinson's disease. Neurobiol Dis. (2006) 21:404–12. doi: 10.1016/j.nbd.2005.08.002 16182554

[20] Fiszer U Mix E Fredrikson S Kostulas V Link H. Parkinson's disease and immunological abnormalities: increase of HLA-DR expression on monocytes in cerebrospinal fluid and of CD45RO+ T cells in peripheral blood. Acta Neurol Scand. (1994) 90:160–6. doi: 10.1111/(ISSN)1600-0404 7847055

[21] Smajić S Prada-Medina CA Landoulsi Z Ghelfi J Delcambre S Dietrich C. Single-cell sequencing of human midbrain reveals glial activation and a Parkinson-specific neuronal state. Brain. (2022) 145:964–78. doi: 10.1093/brain/awab446 PMC9050543 34919646

[22] Tansey MG Wallings RL Houser MC Herrick MK Keating CE Joers V. Inflammation and immune dysfunction in Parkinson disease. Nat Rev Immunol. (2022) 22:657–73. doi: 10.1038/s41577-022-00684-6 PMC8895080 35246670

[23] Saade M Araujo de Souza G Scavone C Kinoshita PF. The role of GPNMB in inflammation. Front Immunol. (2021) 12:674739. doi: 10.3389/fimmu.2021.674739 34054862 PMC8149902

[24] Diaz-Ortiz ME Seo Y Posavi M Cordon MC Clark E Jain N. GPNMB confers risk for Parkinson's disease through interaction with alpha-synuclein. Science. (2022) 377:eabk0637. doi: 10.1126/science.abk0637 35981040 PMC9870036

[25] Kia DA Zhang D Guelfi S Manzoni C Hubbard L Reynolds RH. Identification of candidate Parkinson disease genes by integrating genome-wide association study, expression, and epigenetic data sets. JAMA Neurol. (2021) 78:464. doi: 10.1001/jamaneurol.2020.5257 33523105 PMC7851759

[26] Zhou S Tian Y Song X Xiong J Cheng G. Brain proteome-wide and transcriptome-wide association studies, Bayesian colocalization and Mendelian randomization analyses revealed causal genes of Parkinson's disease. J Gerontol A Biol Sci Med Sci. (2022). doi: 10.1093/gerona/glac245 36511626

[27] International Parkinson's Disease Genomics C Wellcome Trust Case Control C. A two-stage meta-analysis identifies several new loci for Parkinson's disease. PloS Genet. (2011) 7:e1002142. doi: 10.1371/journal.pgen.1002142 21738488 PMC3128098

[28] Hüttenrauch M Ogorek I Klafki H Otto M Stadelmann C Weggen S. Glycoprotein NMB: a novel Alzheimer’s disease associated marker expressed in a subset of activated microglia. Acta Neuropathol Commun. (2018) 6. doi: 10.1186/s40478-018-0612-3 PMC6194687 30340518

[29] Krasemann S Madore C Cialic R Baufeld C Calcagno N Fatimy RE. The TREM2-APOE pathway drives the transcriptional phenotype of dysfunctional microglia in neurodegenerative diseases. Immunity. (2017) 47:566–581.e9. doi: 10.1016/j.immuni.2017.08.008 28930663 PMC5719893

[30] Török N Maszlag-Török R Molnár K Szolnoki Z Somogyvári F Boda K. Single nucleotide polymorphisms of indoleamine 2,3-dioxygenase 1 influenced the age onset of Parkinson's disease. FBL. (2022) 27. doi: 10.31083/j.fbl2709265 36224022

[31] Sayers EW Bolton EE Brister JR Canese K Chan J Comeau DC. Database resources of the national center for biotechnology information. Nucleic Acids Res. (2022) 50:D20–d26. doi: 10.1093/nar/gkab1112 34850941 PMC8728269

[32] Moloney EB Moskites A Ferrari EJ Isacson O Hallett PJ. The glycoprotein GPNMB is selectively elevated in the substantia nigra of Parkinson's disease patients and increases after lysosomal stress. Neurobiol Dis. (2018) 120:1–11. doi: 10.1016/j.nbd.2018.08.013 30149180 PMC6748034

[33] Kaiser S Zhang L Mollenhauer B Jacob J Longerich S Del-Aguila J. A proteogenomic view of Parkinson’s disease causality and heterogeneity. NPJ Parkinson's Dis. (2023) 9:24. doi: 10.1038/s41531-023-00461-9 36774388 PMC9922273

[34] Budge KM Neal ML Richardson JR Safadi FF. Transgenic overexpression of GPNMB protects against MPTP-induced neurodegeneration. Mol Neurobiol. (2020) 57:2920–33. doi: 10.1007/s12035-020-01921-6 32436108

[35] Neal ML Boyle AM Budge KM Safadi FF Richardson JR. The glycoprotein GPNMB attenuates astrocyte inflammatory responses through the CD44 receptor. J Neuroinflamm. (2018) 15:73. doi: 10.1186/s12974-018-1100-1 PMC5842560 29519253

[36] Murthy MN Blauwendraat C Guelfi S Hardy J Lewis PA Trabzuni D. Increased brain expression of GPNMB is associated with genome wide significant risk for Parkinson's disease on chromosome 7p15.3. Neurogenetics. (2017) 18:121–33. doi: 10.1007/s10048-017-0514-8 PMC5522530 28391543

[37] Iwaki H Blauwendraat C Leonard HL Liu G Maple-Grødem J Corvol J-C. Genetic risk of Parkinson disease and progression:: An analysis of 13 longitudinal cohorts. Neurol Genet. (2019) 5:e348. doi: 10.1212/NXG.0000000000000348 31404238 PMC6659137

[38] Chang D Nalls MA Hallgrímsdóttir IB Hunkapiller J van der Brug M Cai F. A meta-analysis of genome-wide association studies identifies 17 new Parkinson's disease risk loci. Nat Genet. (2017) 49:1511–6. doi: 10.1038/ng.3955 PMC5812477 28892059

[39] Nalls MA Pankratz N Lill CM Do CB Hernandez DG Saa M. Large-scale meta-analysis of genome-wide association data identifies six new risk loci for Parkinson's disease. Nat Genet. (2014) 46:989–93. doi: 10.1038/ng.3043 PMC4146673 25064009

[40] Wilson LE Xu Z Harlid S White AJ Troester MA Sandler DP. Alcohol and DNA methylation: an epigenome-wide association study in blood and normal breast tissue. Am J Epidemiol. (2019) 188:1055–65. doi: 10.1093/aje/kwz032 PMC6545285 30938765

[41] Liu C Marioni RE Hedman ÅK Pfeiffer L Tsai P-C Reynolds LM. A DNA methylation biomarker of alcohol consumption. Mol Psychiatry. (2018) 23:422–33. doi: 10.1038/mp.2016.192 PMC5575985 27843151

[42] Zakhari S. Alcohol metabolism and epigenetics changes. Alcohol Res. (2013) 35:6–16. 24313160 10.35946/arcr.v35.1.02 PMC3860421

[43] Philibert RA Plume JM Gibbons FX Brody GH Beach SR. The impact of recent alcohol use on genome wide DNA methylation signatures. Front Genet. (2012) 3:54. doi: 10.3389/fgene.2012.00054 22514556 PMC3322340

[44] Manzardo AM Henkhaus RS Butler MG. Global DNA promoter methylation in frontal cortex of alcoholics and controls. Gene. (2012) 498:5–12. doi: 10.1016/j.gene.2012.01.096 22353363 PMC3653411

[45] Lanquetin A Leclercq S de Timary P Segobin S Naveau M Coulbault L. Role of inflammation in alcohol-related brain abnormalities: a translational study. Brain Commun. (2021) 3:fcab154. doi: 10.1093/braincomms/fcab154 34396111 PMC8361421

[46] Lowe PP Morel C Ambade A Iracheta-Vellve A Kwiatkowski E Satishchandran A. Chronic alcohol-induced neuroinflammation involves CCR2/5-dependent peripheral macrophage infiltration and microglia alterations. J Neuroinflamm. (2020) 17:296. doi: 10.1186/s12974-020-01972-5 PMC7547498 33036616

[47] Orio L Alen F Pavon FJ Serrano A Garcia-Bueno B. Oleoylethanolamide, neuroinflammation, and alcohol abuse. Front Mol Neurosci. (2018) 11:490. doi: 10.3389/fnmol.2018.00490 30687006 PMC6333756

[48] Szabo G Lippai D. Chapter Eleven - Converging Actions of Alcohol on Liver and Brain Immune Signaling. In: Cui C Shurtleff D Harris RA, editors. International Review of Neurobiology. SAN DIEGO, CA: Academic Press (2014). p. 359–80. 10.1016/B978-0-12-801284-0.00011-7 25175869

[49] Harris PS Michel CR Yun Y McGinnis CD Assiri MA Ahmadi AR. Proteomic analysis of alcohol-associated hepatitis reveals glycoprotein NMB (GPNMB) as a novel hepatic and serum biomarker. Alcohol. (2022) 99:35–48. doi: 10.1016/j.alcohol.2021.11.005 34923085 PMC8919678

[50] Hamada K Ferguson LB Mayfield RD Krishnan HR Maienschein-Cline M Lasek AW. Binge-like ethanol drinking activates anaplastic lymphoma kinase signaling and increases the expression of STAT3 target genes in the mouse hippocampus and prefrontal cortex. Genes Brain Behav. (2021):e12729. doi: 10.1111/gbb.12729 33641239 PMC8944393

[51] Shao C Wang X Wang P Tang H He J Wu N. Parkinson's disease risk and alcohol intake: A systematic review and dose-response meta-analysis of prospective studies. Front Nutr. (2021) 8:709846. doi: 10.3389/fnut.2021.709846 34722604 PMC8551485

[52] Peters S Gallo V Vineis P Middleton LT Forsgren L Sacerdote C. Alcohol consumption and risk of Parkinson's disease: data from a large prospective European cohort. Mov Disord. (2020) 35:1258–63. doi: 10.1002/mds.28039 PMC7496254 32357270

[53] Kim IY Yang TO Heath AK Simpson RF Reeves GK Green J. Alcohol intake and Parkinson's disease risk in the million women study. Mov Disord. (2020) 35:443–9. doi: 10.1002/mds.27933 PMC7155013 31769113

[54] Bettiol SS Rose TC Hughes CJ Smith LA. Alcohol consumption and Parkinson's disease risk: A review of recent findings. J Parkinsons Dis. (2015) 5:425–42. doi: 10.3233/JPD-150533 PMC4923726 26406123

[55] Fukushima W Miyake Y Tanaka K Sasaki S Kiyohara C Tsuboi Y. Alcohol drinking and risk of Parkinson's disease: a case-control study in Japan. BMC Neurol. (2010) 10:111. doi: 10.1186/1471-2377-10-111 21054827 PMC2991300

[56] Eriksson AK Löfving S Callaghan RC Allebeck P. Alcohol use disorders and risk of Parkinson's disease: findings from a Swedish national cohort study 1972-2008. BMC Neurol. (2013) 13:190. doi: 10.1186/1471-2377-13-190 24314068 PMC4029307

[57] Chen CH Yang JH Chiang CWK Hsiung C-N Wu P-E Chang L-C. Population structure of Han Chinese in the modern Taiwanese population based on 10,000 participants in the Taiwan Biobank project. Hum Mol Genet. (2016) 25:5321–31. doi: 10.1093/hmg/ddw346 PMC6078601 27798100

[58] Wei C-Y Yang J-H Yeh E-C Tsai M-F Kao H-J Lo C-Z. Genetic profiles of 103,106 individuals in the Taiwan Biobank provide insights into the health and history of Han Chinese. NPJ Genom Med. (2021) 6. doi: 10.1038/s41525-021-00178-9 PMC7878858 33574314

[59] Pringsheim T Jette N Frolkis A Steeves TD. The prevalence of Parkinson's disease: a systematic review and meta-analysis. Mov Disord. (2014) 29:1583–90. doi: 10.1002/mds.25945 24976103

[60] Noyce AJ Bestwick JP Silveira-Moriyama L Hawkes CH Giovannoni G Lees AJ. Meta-analysis of early nonmotor features and risk factors for Parkinson disease. Ann Neurol. (2012) 72:893–901. doi: 10.1002/ana.23687 23071076 PMC3556649

[61] Liu R Guo X Park Y Huang X Sinha R Freedman ND. Caffeine intake, smoking, and risk of Parkinson disease in men and women. Am J Epidemiol. (2012) 175:1200–7. doi: 10.1093/aje/kwr451 PMC3370885 22505763

[62] Chen H Huang X Guo X Mailman RB Park Y Kamel F. Smoking duration, intensity, and risk of Parkinson disease. Neurology. (2010) 74:878–84. doi: 10.1212/WNL.0b013e3181d55f38 PMC2836869 20220126

[63] Quik M. Smoking, nicotine and Parkinson's disease. Trends Neurosci. (2004) 27:561–8. doi: 10.1016/j.tins.2004.06.008 15331239

[64] Du P Zhang X Huang CC Jafari N Kibbe WA Hou L. Comparison of Beta-value and M-value methods for quantifying methylation levels by microarray analysis. BMC Bioinf. (2010) 11:587. doi: 10.1186/1471-2105-11-587 PMC3012676 21118553

[65] Shen L Kondo Y Guo Y Zhang J Zhang L Ahmed S. Genome-wide profiling of DNA methylation reveals a class of normally methylated cpG island promoters. PloS Genet. (2007) 3:e181. doi: 10.1371/journal.pgen.0030181 17967063 PMC2041996

[66] Irizarry RA Ladd-Acosta C Carvalho B Wu H Brandenburg SA Jeddeloh JA. Comprehensive high-throughput arrays for relative methylation (CHARM). Genome Res. (2008) 18:780–90. doi: 10.1101/gr.7301508 PMC2336799 18316654

[67] Rahmani E Zaitlen N Baran Y Eng C Hu D Galanter J. Sparse PCA corrects for cell type heterogeneity in epigenome-wide association studies. Nat Methods. (2016) 13:443–5. doi: 10.1038/nmeth.3809 PMC5548182 27018579

[68] Shi S Yuan N Yang M Du Z Wang J Sheng X. Comprehensive assessment of genotype imputation performance. Hum Hered. (2018) 83:107–16. doi: 10.1159/000489758 30669139

[69] Chang CC Chow CC Tellier LC Vattikuti S Purcell SM Lee JJ. Second-generation PLINK: rising to the challenge of larger and richer datasets. Gigascience. (2015) 4:7. doi: 10.1186/s13742-015-0047-8 25722852 PMC4342193

[70] Graham SE Clarke SL Wu K-HH Kanoni S Zajac GJM Ramdas S. The power of genetic diversity in genome-wide association studies of lipids. Nature. (2021) 600:675–9. doi: 10.1038/s41586-021-04064-3 PMC8730582 34887591

[71] Popejoy AB Fullerton SM. Genomics is failing on diversity. Nature. (2016) 538:161–4. doi: 10.1038/538161a PMC5089703 27734877

[72] Sirugo G Williams SM Tishkoff SA. The missing diversity in human genetic studies. Cell. (2019) 177:26–31. doi: 10.1016/j.cell.2019.02.048 30901543 PMC7380073

[73] Prasad R Jho EH. A concise review of human brain methylome during aging and neurodegenerative diseases. BMB Rep. (2019) 52:577–88. doi: 10.5483/BMBRep.2019.52.10.215 PMC6827576 31462381

[74] Langmyhr M Henriksen SP Cappelletti C van de Berg WDJ Pihlstrøm L Toft M. Allele-specific expression of Parkinson's disease susceptibility genes in human brain. Sci Rep. (2021) 11:504. doi: 10.1038/s41598-020-79990-9 33436766 PMC7804400

[75] Collaborators GBDPsD. Global, regional, and national burden of Parkinson's disease, 1990-2016: a systematic analysis for the Global Burden of Disease Study 2016. Lancet Neurol. (2018) 17:939–53. doi: 10.1016/S1474-4422(18)30295-3 PMC6191528 30287051

[76] Ng Y-F Ng E Lim E-W Prakash KM Tan LCS Tan E-K. Case-control study of hypertension and Parkinson’s disease. NPJ Parkinson's Dis. (2021) 7:63. doi: 10.1038/s41531-021-00202-w 34290246 PMC8295270

[77] Chen J Zhang C Wu Y Zhang D. Association between hypertension and the risk of Parkinson’s disease: A meta-analysis of analytical studies. Neuroepidemiology. (2019) 52:181–92. doi: 10.1159/000496977 30726850

[78] Hou L Li Q Jiang L Qiu H Geng C Hong J-S. Hypertension and diagnosis of Parkinson's disease: A meta-analysis of cohort studies. Front Neurol. (2018) 9:162. doi: 10.3389/fneur.2018.00162 29615961 PMC5867351

[79] Qiu C Hu G Kivipelto M Laatikainen T Antikainen R Fratiglioni L. Association of blood pressure and hypertension with the risk of Parkinson disease. Hypertension. (2011) 57:1094–100. doi: 10.1161/HYPERTENSIONAHA.111.171249 21536985

[80] Paganini-Hill A. Risk factors for Parkinson's disease: the leisure world cohort study. Neuroepidemiology. (2001) 20:118–24. doi: 10.1159/000054770 11359079

[81] McCann SJ LeCouteur DG Green AC Brayne C Johnson AG Chan D. The epidemiology of Parkinson's disease in an Australian population. Neuroepidemiology. (1998) 17:310–7. doi: 10.1159/000026185 9778597

[82] Simmering JE Welsh MJ Schultz J Narayanan NS. Use of glycolysis-enhancing drugs and risk of Parkinson's disease. Mov Disord. (2022) 37:2210–6. doi: 10.1002/mds.29184 PMC9669185 36054705

[83] Lin HC Tseng YF Shen AL Chao JC Hsu CY Lin HL. Association of angiotensin receptor blockers with incident Parkinson disease in patients with hypertension: A retrospective cohort study. Am J Med. (2022) 135:1001–7. doi: 10.1016/j.amjmed.2022.04.029 35580718

[84] Jo Y Kim S Ye BS Lee E Yu YM. Protective effect of renin-angiotensin system inhibitors on Parkinson's disease: A nationwide cohort study. Front Pharmacol. (2022) 13:837890. doi: 10.3389/fphar.2022.837890 35308220 PMC8927987

[85] Simmering JE Welsh MJ Liu L Narayanan NS Pottegård A. Association of glycolysis-enhancing α-1 blockers with risk of developing Parkinson disease. JAMA Neurol. (2021) 78:407–13. doi: 10.1001/jamaneurol.2020.5157 PMC7851758 33523098

[86] Cai R Zhang Y Simmering JE Schultz JL Li Y Fernandez-Carasa I. Enhancing glycolysis attenuates Parkinson’s disease progression in models and clinical databases. J Clin Invest. (2019) 129:4539–49. doi: 10.1172/JCI129987 PMC6763248 31524631

[87] Lee Y-C Lin C-H Wu R-M Lin J-W Chang C-H Lai M-S. Antihypertensive agents and risk of Parkinson's disease: A nationwide cohort study. PloS One. (2014) 9:e98961. doi: 10.1371/journal.pone.0098961 24910980 PMC4049613

[88] Aguet F Anand S Ardlie KG Gabriel S Getz GA Graubert A. The GTEx Consortium atlas of genetic regulatory effects across human tissues. Science. (2020) 369:1318–30. doi: 10.1126/science.aaz1776 PMC7737656 32913098

[89] Huang D Zhou Y Yi X Fan X Wang J Yao H. VannoPortal: multiscale functional annotation of human genetic variants for interrogating molecular mechanism of traits and diseases. Nucleic Acids Res. (2021) 50:D1408–16. doi: 10.1093/nar/gkab853 PMC8728305 34570217

